# Canagliflozin for Japanese patients with chronic heart failure and type II diabetes

**DOI:** 10.1186/s12933-019-0877-2

**Published:** 2019-06-05

**Authors:** Akira Sezai, Hisakuni Sekino, Satoshi Unosawa, Makoto Taoka, Shunji Osaka, Masashi Tanaka

**Affiliations:** 1 Department of Cardiovascular Surgery, Nihon University School of Medicine, 30-1 Oyaguchi-kamimachi, Itabashi-ku, Tokyo, 173-8610 Japan; 2 Sekino Hospital, Tokyo, Japan

**Keywords:** SGLT2 inhibitor, Diabetes, Heart failure

## Abstract

**Background:**

Reports that sodium glucose cotransporter 2 inhibitors decrease cardiovascular death and events in patients with diabetes have attracted attention in the cardiology field. We conducted a study of canagliflozin in patients with chronic heart failure and type II diabetes.

**Methods:**

Thirty-five Japanese patients with chronic heart failure and type II diabetes were treated with canagliflozin for 12 months. The primary endpoints were the changes of subcutaneous, visceral, and total fat areas at 12 months determined by computed tomography. Secondary endpoints included markers of glycemic control, renal function, and oxidative stress, as well as lipid parameters, atrial natriuretic peptide (ANP), brain natriuretic peptide (BNP), flow-mediated dilation (FMD), and echocardiographic left ventricular function.

**Results:**

All fat areas (subcutaneous, visceral, and total) showed a significant decrease at 12 months. ANP and BNP also decreased significantly, along with improvement of renal function, oxidized LDL, and E/e′, FMD increased significantly after canagliflozin treatment.

**Conclusion:**

Canagliflozin demonstrated cardiac and renal protective effects as well as improving oxidative stress, diastolic function, and endothelial function. This drug was effective in patients who had heart failure with preserved ejection fraction and could become first-line therapy for such patients with diabetes.

*Trial registration* UMIN (http://www.umin.ac.jp/), Study ID: UMIN000021239

## Background

The EMPA-LEG-OUTCOME, CANVAS, and DECLERE-TIMI58 trials have demonstrated that sodium glucose cotransporter 2 inhibitors (SGLT2-is) can decrease cardiovascular death, cardiovascular events, and renal events in patients with type II diabetes, attracting strong attention among cardiologists [1–3]. At present, a large-scale study of SGLT2-i therapy is ongoing in patients with chronic heart failure and it expected that these drugs may be able to play a role in heart failure management [4].

SGLT2-is have multiple pharmacological actions and demonstrate protective effects on the heart and kidneys, as well as controlling blood glucose levels in patients with diabetes. When these drugs block reabsorption of glucose and sodium in the proximal renal tubules, an increase of sodium and chloride reaching the macula densa potentiates tubulo-glomerular feedback and promotes contraction of afferent arterioles to suppress excessive glomerular filtration [5]. This helps to decrease albuminuria and prevent deterioration of glomerular filtration. In addition, increased urinary excretion of glucose results in a decrease of fat volume, hemoglobin A1c (HbA1c), uric acid, and systemic inflammation. Furthermore, increased sodium excretion leads to a decrease of the plasma volume and blood pressure, which may promote cardiac and renal protection [5–7]. However, while large-scale clinical studies have apparently demonstrated a lower incidence of cardiac and renal events in patients taking SGLT2-is, the detailed mechanisms underlying these beneficial effects are still unknown. Various pharmacological actions of SGLT2-is have been identified by previous studies. For example, SGLT2-is have been shown to significantly decrease fat volume, potentially leading to weight loss, reduction of HbA1c, and favorable effects on lipid and triglyceride metabolism. It was also reported that a decrease of fat volume may improve oxidative stress, endothelial function, and inflammation. However, no studies have investigated the effects of SGLT2-is on fat volume, oxidative stress, and vascular endothelial function. Accordingly, we performed a study to determine whether a decrease of fat volume with SGLT2-i therapy could improve oxidative stress and endothelial function. Since it was considered that the effects of SGLT2-i therapy might only be detected over the long term, the primary endpoints of the study were assessed at 12 months. Because large-scale clinical studies have already demonstrated that SGLT2-is can significantly reduce the incidence of cardiovascular events, cardiac function (including diastolic function) was also evaluated at 12 months.

## Methods

### Study protocol

This study was a prospective controlled trial of canagliflozin in outpatients with chronic heart failure and diabetes (CANOSSA trial: prospective, open-label, add-on trial of canagliflozin for diabetes mellitus and stable chronic heart failure). The subjects were stable outpatients with chronic heart failure and type II diabetes mellitus. Stable outpatients were defined as patients whose oral medications had not been changed for 6 months, while patients with chronic heart failure were defined as those with a history of heart failure who were currently on oral medications for treatment of heart failure (diuretics, β-blockers, and renin-angiotensin system inhibitors). Patients received add-on treatment with canagliflozin (Mitsubishi Tanabe Pharma Co., Osaka, Japan and Daiichi Sankyo Company, Ltd., Tokyo, Japan) at 100 mg/day for 12 months.

During the study period, other medications were adjusted at the discretion of the investigators to manage worsening of heart failure and edema or changes of blood pressure. Exclusion criteria were as follows: (1) renal dysfunction with an estimated glomerular filtration rate (eGFR) < 30 mL/min/1.73 m^2^, (2) hepatic dysfunction, (3) pregnancy, and (4) patients who were judged to be unsuitable for other reasons by the attending physician. This study was conducted at Sekino Hospital, which is a peripheral hospital of Nihon University Itabashi Hospital. The details of the study were explained to the patients before enrollment and informed consent was obtained. Approval of our institutional review board was also obtained and the study was registered with the University hospital Medical Information Network (study ID: UMIN000021239).

### Endpoints

The primary endpoints were the areas of subcutaneous fat, visceral fat, and total fat determined by computed tomography (CT) (VCT, GE Healthcare Japan, Inc., Tokyo, Japan). Patients fasted on the day of CT scanning, and images were obtained at the level of the umbilicus with a slice thickness of 10 mm and standard scale. Then the fat areas were measured on CT scans by using special software (Fat Scan Premium Ver. 5.0; East Japan Institute of Technology Co., Ltd., Ibaraki, Japan).

The secondary endpoints included body weight (BW), body mass index (BMI), biomarkers of diabetes [HbA1c, insulin, and C-peptide immunoreactivity (CPR)], renal function parameters [blood urea nitrogen (BUN), serum creatinine (s-Cr), eGFR, cystatin-C, and urinary albumin excretion adjusted for urinary creatinine (U-alb)], and lipid and fat-related parameters [total cholesterol (T-cho), low-density lipoprotein cholesterol (LDL), high-density lipoprotein cholesterol (HDL), nonHDL cholesterol (nonHDL = T-cho-HDL), triglyceride (TG), remnant-like particles cholesterol (RLP-cho), and the eicosapentaenoic acid/arachidonic acid ratio (EPA/AA ratio)]. We also measured hemoglobin (Hb), hematocrit (Ht), atrial natriuretic peptide (ANP), brain natriuretic peptide (BNP), oxidized low density lipoprotein (Ox-LDL), high-sensitivity C-reactive protein (hs-CRP), plasma renin activity (renin), angiotensin-II, aldosterone, and urinalysis parameters (sugar and ketone bodies). Furthermore, we measured the pulse wave velocity (PWV), flow-mediated dilation (FMD), and echocardiography parameters [left ventricular end-diastolic diameter (LVDd), left ventricular end-systolic diameter (LVDs), ejection fraction (EF), percent fractional shortening (%FS), E/e′ ratio by pulsed wave Doppler, and left ventricular mass index (LVMI)], as well as assessing adverse reactions. BW, BMI, BUN, s-Cr, eGFR, T-cho, LDL, HDL, nonHDL, TG, Hb, Ht, ANP, BNP, hs-CRP, and urinalysis parameters were measured before initiation of canagliflozin treatment and after 1, 3, 6, and 12 months of treatment, while diabetes biomarkers, cystatin-C, U-alb, RLP-cho, Ox-LDL, EPA/AA ratio, ANP, BNP, renin, angiotensin-II, and aldosterone were measured before starting canagliflozin treatment and after 3, 6, and 12 months of treatment. Assessment of PWV (BP-203RPE, Omron Healthcare Co., Ltd., Kyoto, Japan) and FMD (EF38G, UNEX Corporation, Nagoya, Japan), as well as echocardiography (LOGIQ S8, GE Healthcare Japan Corp., Tokyo, Japan), were performed before canagliflozin treatment and after 6 and 12 months of treatment. For measurement of PWV, sphygmomanometer cuffs with pulse wave sensors were applied to the upper arms and ankles bilaterally. The electrocardiogram, heart sounds, and pulse wave were measured at the same time. The blood vessel length between measurement points was calculated automatically from the height entered into the instrument, and was divided by pulse rate transmission time between the measurement points to determine the PWV. For measurement of FMD, patients were instructed to avoid exercise for 6 h prior to testing, as well as avoiding intake of caffeine, high-fat meals, vitamin C, and smoking. With the patient resting supine on a bed, a cuff was applied to the left forearm and ECG electrodes were attached to both wrists. An ultrasound probe was applied to the left upper arm to visualize the brachial artery at rest. Then the cuff on the forearm was inflated for 5 min at the mean systolic blood pressure + 50 mmHg. After release of the cuff, data on the vessel diameter were obtained and graphed over time. Next, the maximum diameter after release of the cuff was subtracted from the resting diameter to obtain FMD. Adverse reactions were classified as follows: renal dysfunction (≥ 50% increase of serum Cr), hepatic dysfunction (≥ 50% increase of AST or ALT), gastrointestinal symptoms, infection, dehydration, and allergic reactions. Management of adverse reactions (discontinuation of canagliflozin, etc.) was determined by the attending physician. During the study period, oral medications were not adjusted, if possible. However, patients were expected to maintain a systolic blood pressure of 100–140 mmHg and heart rate of 50–90/min at early morning home measurement, so antihypertensive drugs were added, decreased in dose, or discontinued as required. When an antihypertensive drug was added, decreased in dose, or discontinued, it was preferentially a calcium antagonist that was added, decreased, or discontinued. For heart rate control, a ß-blocker was added, decreased in dose, or discontinued. Investigators were also allowed to adjust diuretic therapy for dehydration or in response to worsening/improvement of systemic and lower limb edema.

### Statistical analysis

Results are expressed as the mean ± standard error of the mean (SEM). One-way analysis of variance (ANOVA) was used to compare parameters and p < 0.05 was considered to indicate statistical significance. All analyses were conducted with SPSS software (SPSS Inc., Illinois). Data aggregation was conducted by Sekino Laboratory staffs not involved in this study, statistical analysis was conducted by SATISTA (Kyoto, Japan), a company not involved in the conduct of this study.

## Results

### Patients

Thirty-five patients were enrolled in this trial and their baseline characteristics are shown in Table 1. There were no dropouts and all patients completed the 1-year study period without complications.

**Table 1:** Patient characteristics

Number	35
Age (years)	71.4 ± 11.3
Gender (male: female)	25: 7
Cause of heart failure	
Hypertension	17 (49%)
Ischemia	9 (26%)
Valve disease	6 (17%)
Cardiomyopathy	3 (8%)
Risk factors	
Hypertension	27 (77%)
Dyslipidemia	29 (83%)
Hyperuricemia	19 (54%)
Chronic kidney disease	24 (69%)
Obesity	18 (51%)
Metabolic syndrome	19 (54%)
Past smoker	13 (37%)
Current smoker	0
Classification of heart failure	
HFrEF	2 (6%)
HFpEF	33 (94%)
Medication	
Oral hypoglycemic agent	
Alpha-glucosidase inhibitor	5 (14%)
Biguanide	5 (14%)
DPP4 inhibitor	16 (46%)
Sulfonylurea	9 (26%)
Thiazolidine derivatives	1 (3%)
No medication	15 (43%)
ACE-inhibitor	2 (6%)
Aldosterone blocker	16 (46%)
ARB	13 (37%)
Beta blocker	28 (80%)
Calcium antagonist	10 (29%)
Renin-inhibitor	1 (3%)
Furosemide	13 (37%)
Tolvaptan	1 (3%)
Statin	28 (80%)
XO inhibitor	17 (49%)

*HFrEF* heart failure with reduced ejection fraction, *HFpEF* heart failure with preserved ejection fraction, *DDP* dipeptidyl peptidase, *ARB* angiotensin II receptor blocker, *ACE* angiotensin-converting enzyme, *XO* xanthine oxidase

### Primary endpoint (Figs. 1 and 2)

Compared with the values before treatment (baseline), all three fat areas (subcutaneous fat area, visceral fat area, and total fat area) showed a significant decrease at 6 months and 12 months after initiation of canagliflozin treatment (all p < 0.001).

**Fig. 1:**
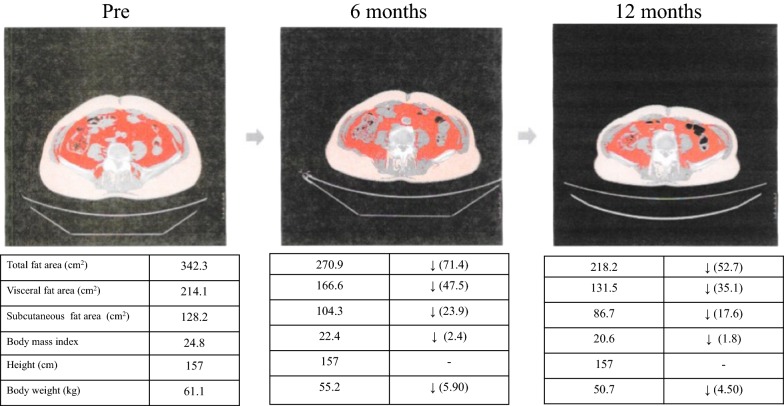
A case of each fat areas, body mass index, and body weigh changes

**Fig. 2:**
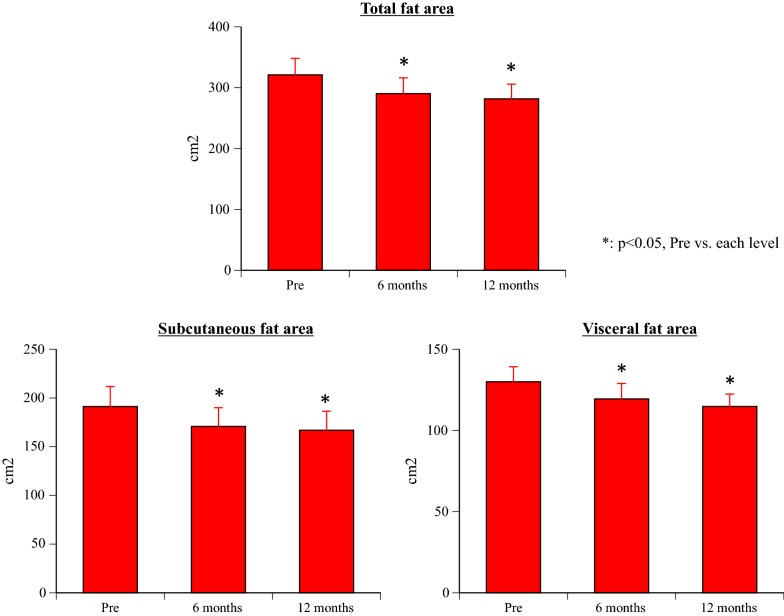
Changes of the total fat area, subcutaneous fat area, and visceral fat area

### Secondary endpoints

#### BW, BMI, and metabolic syndrome (Table 2)

BW and BMI showed a significant decrease at 6 months and 12 months after the start of canagliflozin treatment compared with baseline (all p < 0.001). Although BW showed a further significant decrease at 12 months compared with 6 months (p = 0.046), there was no significant difference of BMI between 6 months and 12 months (p = 0.081). The prevalence of metabolic syndrome was significantly lower at 12 months compared with before treatment (p = 0.0134).

**Table 2:** Changes of each blood and urine test

	Pre	1 month	3 months	6 months	12 months
Body weight (kg)	66.7 ± 3.6	–	–	64.7 ± 3.5*	63.8 ± 3.5*^#^
Body mass index	25.6 ± 1.1	–	–	24.7 ± 1.0*	24.3 ± 1.0*
Metabolic syndrome	19 (54%)	–	–	10 (29%)	8 (23%)*
Hemoglobin (g/dL)	12.5 ± 0.4	12.6 ± 0.4	12.7 ± 0.4	12.8 ± 0.4	13.3 ± 0.4*^$+^
Hematocrit (%)	38.0 ± 1.1	38.4 ± 1.2	38.3 ± 1.2	38.6 ± 1.2	40.7 ± 1.2*^$+#^
HbA1c (%)	6.61 ± 0.20	6.51 ± 0.21*	6.52 ± 0.19	6.31 ± 0.19*^$+^	6.29 ± 0.19*^$+^
Insulin (μlU/mL)	13.8 ± 3.2	–	18.3 ± 5.4	12.9 ± 2.5	13.8 ± 3.2
CPR (ng/mL)	4.21 ± 0.35	–	4.36 ± 0.48	4.00 ± 0.39	3.47 ± 0.29^+^
Blood urea nitrogen (mg/dL)	22.0 ± 1.5	22.2 ± 1.5	22.5 ± 1.3	22.0 ± 1.4	20.8 ± 1.2
Total cholesterol (mg/dL)	147.4 ± 6.1	148.0 ± 6.2	147.4 ± 5.9	145.3 ± 5.8	143.1 ± 5.3
LDL (mg/dL)	82.4 ± 4.8	82.5 ± 4.1	82.4 ± 4.0	79.5 ± 4.1	70.4 ± 3.9*^$+#^
HDL (mg/dL)	52.6 ± 2.5	54.2 ± 2.5*	54.4 ± 2.4*	55.5 ± 2.4*	59.2 ± 3.0*^$+#^
nonHDL (mg/dL)	96.9 ± 4.6	95.8 ± 4.6	95.0 ± 4.1	91.9 ± 4.1	86.1 ± 3.5*^$+^
Triglyceride (mg/dL)	159.2 ± 19.0	122.5 ± 11.5*	111.7 ± 10.0*	108.2 ± 11.4*	107.7 ± 10.2*^$^
RLP-cho (mg/dL)	5.89 ± 0.94	–	4.56 ± 0.58*	4.48 ± 0.71*	3.69 ± 0.62*^+#^
EPA/AA	0.52 ± 0.07	–	0.53 ± 0.07	0.55 ± 0.06	0.57 ± 0.06
hs-CRP (hs-CRP)	0.39 ± 0.07	0.27 ± 0.06	0.20 ± 0.05*	0.20 ± 0.04*	0.21 ± 0.04*
Renin (pg/mL)	10.3 ± 3.9	–	29.6 ± 8.5*	44.4 ± 10.7*	50.7 ± 16.6*
Angiotensin-II (pg/mL)	30.0 ± 5.8	–	29.1 ± 5.2	20.2 ± 3.3*^+^	23.1 ± 5.7
Aldosterone (pg/mL)	219.6 ± 30.3	–	177.9 ± 24.8*	171.3 ± 21.9*	169.2 ± 24.2*
Urine sugar	0.46 ± 0.19	3.63 ± 0.12	3.57 ± 0.12	3.46 ± 0.12	3.46 ± 0.12
Urine ketone	0	0.03 ± 0.03	0	0	0.06 ± 0.04

*HbA1c* hemoglobin A1c, *CPR* C-peptide immunoreactivity, *LDL* low density lipoprotein, *HDL* high density lipoprotein, *RLP-cho* remnant like particles-cholesterol, *EPA* eicosapentaenoic acid, *AA* arachidonic acid, *hs-CRP* high-sensitivity C-reactive protein

* *p *< 0.05, pre vs. each level, ^$^ *p *< 0.05, 1 month vs. each level, ^+^ *p *< 0.05, 3 months vs. each level, ^#^ *p *< 0.05, 6 months vs. each level

#### Hb and Ht (Table 2)

After 12 months of canagliflozin treatment, Hb was significantly increased compared with baseline or after 1 and 3 months of treatment (baseline: p = 0.004, 1 month: p = 0.008, 3 months: p = 0.014). In addition, Ht was significantly increased after 12 months of treatment compared with baseline and other times during the treatment period (baseline: p < 0.001, 1 month: p = 0.001, 3 months: p = 0.001, 6 months: p = 0.004).

#### Diabetes biomarkers (Table 2)

Compared with the baseline value, HbA1c displayed a significant decrease after 1, 6, and 12 months of canagliflozin treatment (1 month: p = 0.022, 6 and 12 months: p < 0.0001). Significant differences were also observed at 6 and 12 months versus 1 month (6 months: p = 0.015, 12 months: p = 0.007) and at 6 and 12 months versus 3 months (6 months: p < 0.0001, 12 months: p < 0.0001). Insulin and glycated albumin did not change significantly after initiation of canagliflozin treatment, but CPR decreased significantly between 3 months and 12 months (p = 0.028).

#### Renal parameters (Table 2 and Fig. 3)

BUN did not change significantly after the initiation of canagliflozin treatment. However, s-Cr showed a significant decrease after 12 months of treatment compared with baseline and after 1, 3, or 6 months of treatment (baseline: p = 0.016, 1 month: p = 0.014, 3 months: p = 0.004, 6 months: p = 0.043). eGFR was significantly increased after 6 and 12 months of treatment compared with baseline (6 months: p = 0.034, 12 months: p = 0.027), while cystatin-C was significantly decreased after 3 and 12 months (3 months: p = 0.047, 12 months: p = 0.048). U-alb showed a significant decrease after 6 months of canagliflozin treatment compared with baseline (p = 0.049), and there was almost a further significant decrease after 12 months (p = 0.053).

**Fig. 3:**
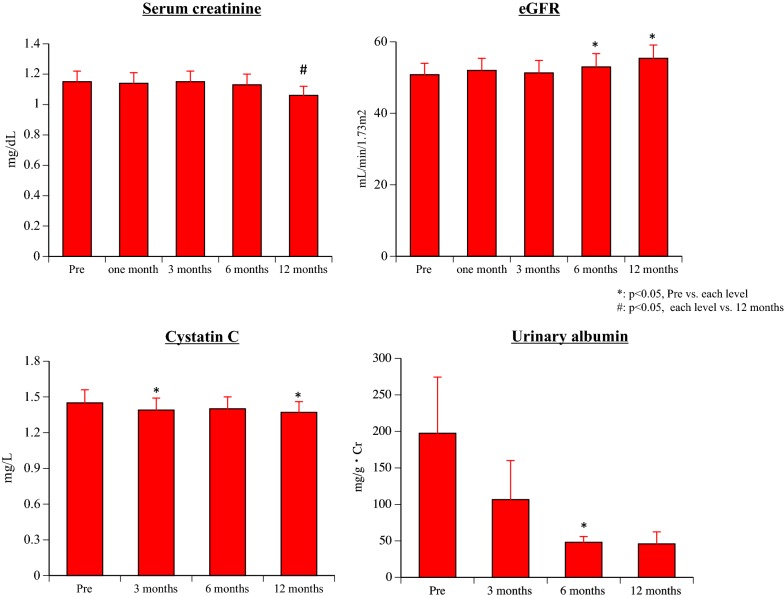
Changes of renal parameters

#### Lipid and fat parameters (Table 2)

Although there was no significant change of T-cho, LDL decreased significantly after 12 months of canagliflozin treatment compared with baseline and compared with after 1, 3, 6 and months of treatment (baseline: p = 0.005, 1 month: p = 0.001, 3 months: p = 0.003, 6 months: p = 0.013). HDL increased significantly after initiation of canagliflozin treatment compared with baseline (1 month: p = 0.041, 3 month: p = 0.038, 6 months: p = 0.004, 12 months: p < 0.001) and also showed a significant increase at 12 months versus 1, 3, and 6 months (1 month: p < 0.001, 3 months: p = 0.001, 6 months: p = 0.007). After 12 months of canagliflozin treatment, nonHDL was significantly lower than at baseline and also compared with 1 and 3 months (baseline: p = 0.017, 1 month: p = 0.029, 3 months: p = 0.044).

TG was significantly decreased by canagliflozin treatment compared with baseline (all p < 0.001) and was also significantly lower at 12 months versus 1 month (p = 0.034). RLP-cho was significantly decreased compared with baseline at 3 months (p = 0.011), 6 months (p = 0.021), and 12 months (p = 0.001) of treatment, and was also significantly decreased at 12 months versus 3 and 6 months (3 months: p = 0.034, 6 months: p = 0.018). The EPA/AA ratio showed no significant changes.

#### ANP and BNP (Fig. 4)

After the start of canagliflozin treatment, ANP was significantly decreased compared with baseline at all times of assessment (1 month: p = 0.001, 3 months: p = 0.0001, 6 months: p < 0.0001, 12 months: p = 0.0001), and also showed a significant decrease at 3, 6, and 12 months versus 1 month (3 months: p = 0.031, 6 months: p = 0.012, 12 months: p = 0.012). Likewise, BNP was significantly decreased by canagliflozin treatment compared with baseline (all p < 0.0001), and was also significantly decreased at 6 and 12 months versus 1 month (6 months: p = 0.003 months: p = 0.002).

**Fig. 4:**
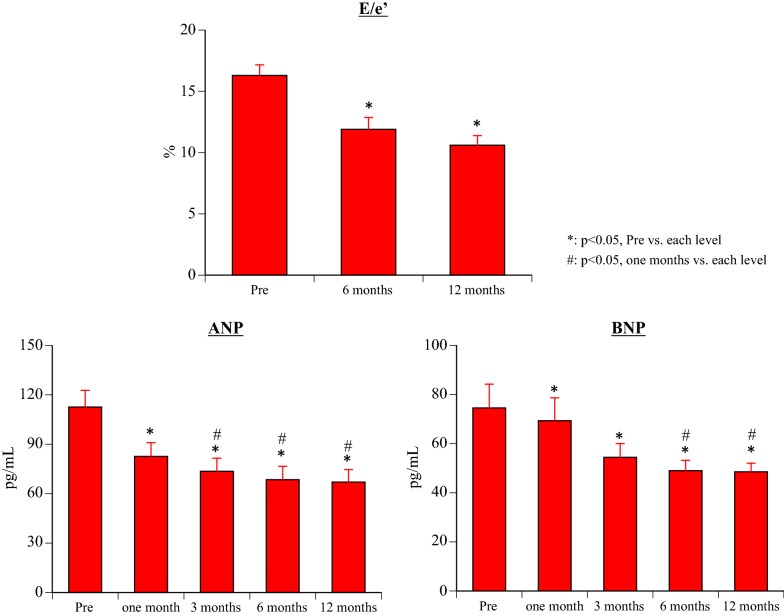
Changes of atrial natriuretic peptide, brain natriuretic peptide, and the ratio of the trans-mitral early peak velocity (E) to e′

#### O-LDL (Fig. 5)

After the start of canagliflozin treatment, there was a significant decrease of O-LDL at all times compared with baseline (all p < 0.0001), as well as a significant decrease at 12 months versus 3 and 6 months (both p = 0.001).

**Fig. 5:**
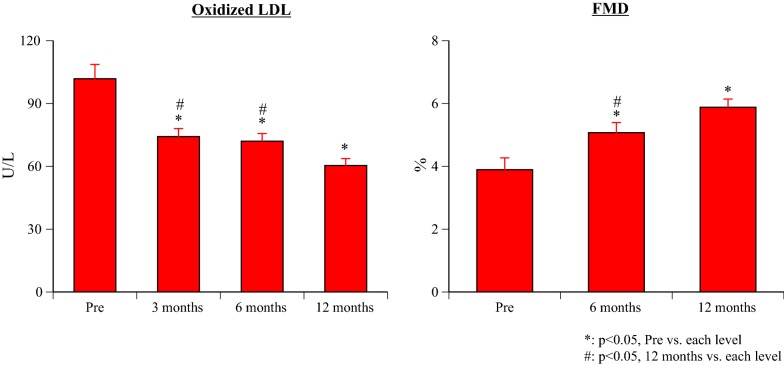
Changes of oxidized low density lipoprotein and flow mediated dilation

#### hs-CRP (Table 2)

There was a significant decrease of hs-CRP after 3, 6, and 12 months of canagliflozin treatment compared with baseline (3 months: p = 0.002, 6 months: p = 0.001, 12 months: p = 0.007).

#### Renin–angiotensin–aldosterone system (Table 2)

Plasma renin activity was significantly increased from 3 months after initiation of treatment compared with baseline (3 months: p = 0.01, 6 months: p = 0.001, 12 months: p = 0.022), while angiotensin-II was significantly decreased at 6 months versus baseline and 3 months (baseline: p = 0.046, 3 months: p = 0.009). Aldosterone showed a significant decrease from 3 months of treatment relative to baseline (3 months: p = 0.005, 6 months: p = 0.011, 12 months: p < 0.001).

#### Urinalysis (Table 2)

Compared with the baseline, urine sugar was significantly increased at all times after initiation of canagliflozin treatment (all p < 0.0001). Urine ketones were 1+ at 1 month and 12 months (only 1 patient was 1+ at 12 months), but there was no significant difference between baseline and the other times of evaluation.

#### PWV (Table 3)

Both systolic and diastolic blood pressure were significantly decreased after 6 months of canagliflozin treatment compared with baseline (systolic: p = 0.001, diastolic: p = 0.018), but the heart rate showed no significant changes. PWV showed a significant decrease bilaterally after 6 months of treatment (right: p = 0.001, left: p < 0.001), but there were no significant changes of right or left ABI at any time.

**Table 3:** Changes of the blood pressure, heart rate, PVW, ABI, echocardiographic data

	Pre	6 months	12 months
S-BP (mmHg)	136.2 ± 2.7	127.5 ± 3.0*	130.1 ± 3.2
D-BP (mmHg)	76.9 ± 1.2	72.7 ± 1.7*	75.2 ± 1.9
Heart rate (/min)	66.9 ± 1.6	65.5 ± 2.2	68.2 ± 1.3
Right PWV (cm/s)	1821.7 ± 70.6	1713.9 ± 64.0*	1784.1 ± 67.0
left PWV (cm/s)	1883.1 ± 82.5	1715.2 ± 69.4*	1814.4 ± 76.7
Right ABI	1.15 ± 0.02	1.14 ± 0.02	1.12 ± 0.03
Left ABI	1.15 ± 0.02	1.14 ± 0.02	1.12 ± 0.02
Echocardiography			
LVDd (mm)	47.1 ± 1.1	45.7 ± 0.9	46.3 ± 1.0
LVDs (mm)	31.6 ± 0.8	30.4 ± 0.7*	29.9 ± 0.8*
Ejection fraction (%)	60.9 ± 1.6	61.9 ± 1.5	64.6 ± 1.3*^$^
% fractional shortening (%)	33.0 ± 0.9	33.4 ± 1.0	35.4 ± 0.9*^$^
LVMI (g/m^2^)	166.5 ± 6.4	147.7 ± 5.8*	140.6 ± 5.4*^$^

*S-BP* systolic blood pressure, *D-BP* diastolic blood pressure, *PWV* pulse power velocity, *ABI* ankle–brachial pressure index, *LVDd* left ventricular end-diastolic diameter, *LVDs* left ventricular end-systolic diameter, *LVMI* left ventricular mass index

* *p *< 0.05, pre vs. each level, ^$^ *p *< 0.05, 6 months vs. 12 months

#### FMD (Fig. 4)

Compared with baseline, FMD was significantly increased at all times after the start of canagliflozin treatment (all p < 0.001), and also showed a significant increase at 12 months versus 6 months (p = 0.001).

#### Echocardiography parameters (Table 3 and Fig. 3)

Two patients (6%) had heart failure with reduced ejection fraction (HFrEF) and 33 patients (94%) had heart failure with preserved ejection fraction (HFpEF). Although LVDd did not change significantly, LVDs was significantly decreased after 6 and 12 months of canagliflozin treatment compared with baseline (6 months: p = 0.027, 12 months: p = 0.016). EF displayed a significant increase at 12 months relative to baseline and 6 months (baseline: p = 0.005, 6 months: p = 0.023), while the E/e′ ratio was significantly decreased at 6 and 12 months versus baseline (both p < 0.001). Finally, LVMI was significantly decreased after 6 and 12 months of treatment compared with baseline (6 months: p = 0.003, 12 months: p < 0.001), and also showed a significant decrease at 12 months relative to 6 months (p = 0.028).

#### Adverse reactions

During the treatment period, there were no adverse events such as hypoglycemia, aggravation of diabetes, dehydration, worsening of heart failure, urinary tract infection, or lower extremity amputation. Three patients had mild lower limb edema and furosemide was discontinued in 4 patients (at 2 months in 2 patients and at 4 months in 2 patients). Antihypertensive drugs were discontinued in 4 patients (Ca antagonists in 1 patient at 5 months and in 3 patients at 6 months; renin inhibitor in 1 patient at 4 months), while β-blockers were discontinued in 3 patients due to a decrease of the heart rate (1 patient at 3 months and 2 patients at 4 months).

## Discussion

**Fig. 6:**
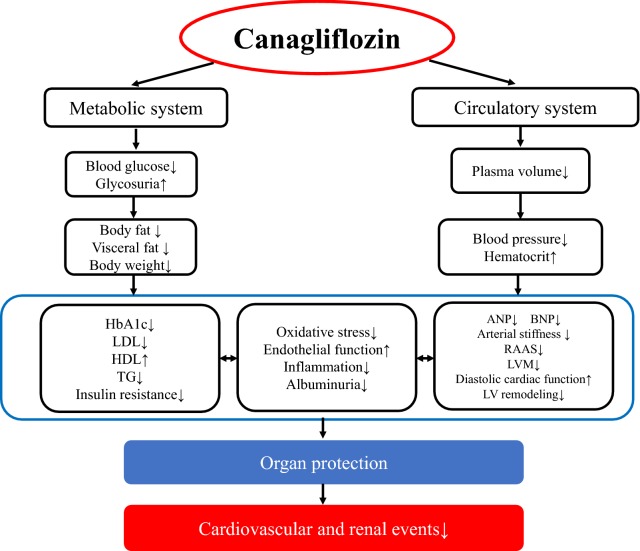
Pharmacological effects of canagliflozin suggested by the findings of this study. *HbA1c* hemoglobin A1c, *LDL* low-density lipoprotein cholesterol, *HDL* high-density lipoprotein cholesterol, *TG* triglyceride, *ANP* atrial natriuretic peptide, *BNP* brain natriuretic peptide, *RAAS* renin–angiotensin–aldosterone system, *LVM* left ventricular mass, *LV* left ventricular

The CANVAS study investigated the efficacy of long-term canagliflozin therapy in 10,142 patients with type 2 diabetes and high cardiovascular risk. After treatment for a mean of 188.2 weeks (median: 126.1 weeks), the canagliflozin group showed lower cardiovascular risk compared to the placebo group, along with slower progression of albuminuria and a lower incidence of renal events [2]. Patients in the CANVAS study were not restricted to those with heart failure and 65.6% had a history of cardiovascular disease. In contrast, the sample size of the present study was small and the observation period was only 12 months, but the subjects were restricted to patients with diabetes and chronic heart failure. In our study, canagliflozin improved cardiac function and renal function, as well as having pleiotropic effects such as improvement of oxidative stress and endothelial function. Efficacy of canagliflozin for decreasing the fat volume, improving oxidative stress, and improving endothelial function was noted soon after initiation of treatment. The cardiac and renal protective effects of this drug were also observed soon after starting treatment. These findings were considered to support the long-term reduction in the incidence of cardiovascular and renal events shown by the CANVAS study (Fig. 6).

Weight loss and reduction of fat volumes were observed after 6 months of canagliflozin treatment and this improvement persisted at 12 months. A previous small-scale study that enrolled 24 patients revealed body weight reduction by a mean of 3 kg after 12 weeks of ipragliflozin treatment, with 70% of this decrease being ascribed to fat loss (40% due to reduction of visceral fat) [8]. The canagliflozin CNATATA-SU study investigated 70 patients, revealing a decrease of visceral fat and subcutaneous fat by 7.3% and 5.4%, respectively, after 52 weeks [9]. The present study was limited to 35 patients, but we also demonstrated a decrease of body weight and body fat with canagliflozin treatment, and the reduction of fat volumes was possibly related to improvement of LDL, HDL, nonHDL, TG, and blood pressure. Thus, although canagliflozin shows low selectivity for SGLT2, reduction of fat volumes was shown by the present investigation and other studies. A decrease of fat volume was also reported in patients treated with another SGLT-i [8]. This suggests that reduction of fat volume is a class effect of SGLT-i therapy. However, no clinical study has investigated the effect on fat volumes from the perspective of SGLT2 selectivity, so this should be clarified in the future.

Many reports have indicated that HbA1c is reduced by SGLT2-is, and we also found a significant decrease in this study. However, the actual reduction was only about 5%, possibly because HbA1c was generally well controlled prior to initiation of canagliflozin [HbA1c was ≥ 8.0% in only 3 patients and was 6.3–6.9% in 27 patients (77%)]. In this study, the insulin level was not affected by canagliflozin therapy and no patient experienced hypoglycemia. Also, CPR was decreased at 12 months, suggesting improvement of insulin resistance, although CPR alone is not sufficient to evaluate either insulin sensitivity or insulin secretion. Accordingly, homeostasis model assessment and steady state plasma glucose monitoring should be performed in future studies.

Almost half of our patients (n = 16, 45%) were not on antidiabetic drugs before the study and received canagliflozin as initial therapy. Therefore, the results suggest that canagliflozin could be a first-line treatment for patients with mild diabetes complicated by chronic heart failure.

It has been reported that reduction of glucose uptake into proximal tubular cells by SGLT2-is markedly decreases oxidative stress [10, 11], but inhibition of oxidative stress has only been found in animal studies before and it seems important that this effect was observed soon after initiation of canagliflozin treatment in the present study. FMD is an index of endothelial function. There has only been 1 report about the effect of SGLT2-is on FMD, which was a study that revealed significant improvement of FMD in patients with HbA1c ≥ 7.0 after 16 weeks of dapagliflozin treatment versus metformin. An oxidative stress marker (urinary 8-OHdG) was also significantly improved by dapagliflozin and it was concluded that alleviation of oxidative stress might have contributed to improvement of FMD [12]. An animal study has shown that attenuation of oxidative stress prevents development of endothelial dysfunction during hyperglycemia [13]. In this study, an oxidative stress marker (ox-LDL) showed a significant decrease at 3 months and then declined further over time, while FMD also decreased over the study period. Inhibition of oxidative stress and improvement of endothelial dysfunction could have positive effects on many organs, leading to a better long-term prognosis.

There have been many reports about renal protection by SGLT2-is, including improvement of albuminuria, maintenance of sCr, and postponement of the need for renal replacement therapy [14, 15]. The present study demonstrated a decrease of sCr after 12 months of canagliflozin therapy and an increase of eGFR from 6 months. In addition, albuminuria was reduced and cystatin C (a sensitive index of glomerular filtration) was significantly decreased, suggesting that canagliflozin improved the glomerular filtration rate. There have been no previous reports about the effects of SGLT2-I therapy on cystatin C. The decrease of albuminuria was probably related to a decrease of intra-glomerular pressure via tubulo-glomerular feedback rather than improvement of blood glucose, since inhibition of albuminuria was not dose-dependent [10].

Regarding the effects of canagliflozin on the heart, ANP and BNP both decreased after initiation of treatment. Thus, cardiac stress was alleviated by canagliflozin, with improvement of diastolic dysfunction and a decrease of LVM also being observed. LVM tends to increase in patients with diabetes and this change is regarded as a risk factor for cardiovascular disorders such as heart failure and sudden death. The effects of oral antidiabetic agents on LVM have not been consistent in previous studies. Ida et al. [11] conducted a meta-analysis of 11 RCTs to investigate changes of LVM following treatment with various agents, including sulfonylureas (gliclazide and glyburide), an α-glucosidase inhibitor (voglibose), metformin, thiazolidinediones (pioglitazone and rosiglitazone), and a DPP-4 inhibitor (sitagliptin), and reported that only gliclazide decreased LVM. There have only been 2 reports about the effects of SGLT2-i therapy on cardiac weight. Matsutani et al. reported that LVM was decreased at 3 months in 37 patients on treatment with canagliflozin, while Soga et al. reported that it was decreased at 6 months in 58 patients receiving dapagliflozin [16, 17].

A prospective study into the influence of SGLT2-i therapy on cardiac weight is ongoing and the results are awaited [18–20]. Many authors have reported improvement of BNP by SGLT2-is, and a decrease of pericardial fat volume was found [21, 22]. The present study suggested that canagliflozin might accelerate reverse remodeling from early after initiation of treatment. Matutani et al. found a decrease of E/e′ at 3 months in their study of canagliflozin and Soga et al. reported a decrease at 6 months in their patients on dapagliflozin [16, 17] Matutani et al. also reported that the decrease of E/e′ was larger in patients with improvement of hemoglobin [16]. An animal study of empagliflozin suggested that an improvement of passive myofilament stiffness and myocardial insulin levels might lead to better diastolic function [23, 24]. In our study, only 2 patients had HFrEF and 33 patients (94%) had HFpEF. While efficacy for HFrEF has frequently been reported for mineralocorticoid receptor antagonists, β-blockers, and ACE inhibitors, effective drugs for HFpEF have not been available.

The ADECLARE-TIMI 58 study demonstrated that dapagliflozin reduced hospitalization for heart failure in patients with or without HFrEF, and also reduced cardiovascular death and ACM in HFrEF patients [25].

These results and our findings suggest that canagliflozin might be useful for HFpEF, but this issue needs further investigation.

SGLT2-is were reported to have a minimal influence on the RAAS. Ninomiya et al. found that renin was increased following treatment with ipragliflozin for 24 weeks, but the change was not significant (p = 0.08). On the other hand, aldosterone increased significantly, possibly to compensate for dehydration due to SGLT2-i therapy [26]. Although they did not discuss the relationship between the RAAS and blood pressure, Ninomiya et al. demonstrated a decrease of blood pressure and PWV during ipragliflozin treatment. Tanaka et al. monitored changes of ANP, NT-pro BNP, and renin for 5 days after initiation of canagliflozin. While ANP and NT-pro BNP both decreased, they found that renin increased, possibly as compensatory mechanism for sodium retention that led to subsequent recovery of urine output [27]. The group of Nishiyama et al. reported that SGLT2 inhibitors frequently cause polyuria and natriuresis, which potentially activate the RAAS in the early stages of treatment. Nevertheless, the effects of SGLT2 inhibitors on RAAS activity are not straightforward. Available data indicate that treatment with SGLT2 inhibitors transiently activates the systemic RAAS in type 2 diabetic patients, but not the intrarenal RAAS [28]. In our study, renin was increased and aldosterone was decreased, contrary to the previous report. Despite, these changes, we noted a decrease of blood pressure and PWV, with the reduction of PWV presumably depending on that of blood pressure. The increase of renin and decrease of aldosterone observed in our study are common during angiotensin-converting-enzyme inhibitor (ACE-I) and angiotensin II receptor blocker (ARB) therapy, but there were no changes of ACE-I and ARB dosages after initiation of canagliflozin. One patient stopped taking a renin inhibitor and 3 patients discontinued furosemide during the study period. It is possible that such changes of medication affected the RAAS. SGLT2-is cause contraction of the afferent arterioles and influence glomerular function, so it would be interesting to determine whether the blood pressure lowering effect of these drugs is mediated by the RAAS.

In addition to the previously reported decrease of HbA1c and body fat volumes, this study demonstrated new findings in patients receiving canagliflozin therapy, such as inhibition of oxidative stress, improvement of endothelial dysfunction, improvement of cardiac diastolic dysfunction, and a decrease of LVM. These effects were noted soon after initiation of treatment. Thus, canagliflozin is an effective antidiabetic agent that may also be useful for prevention and treatment of heart failure and protection of vital organs.

## Limitations

The sample size was of the present study small, it was conducted at a single site, and the observation period was only 1 year. Accordingly, a large-scale multicenter study with a longer follow-up period is required to confirm our findings.

It would have been ideal to enroll several hundred patients in this study. However, although previous small-scale studies have demonstrated that SGLT2-is can decrease fat volume, the effects of these drugs on oxidative stress and endothelial function are still not clearly understood. Accordingly, we conducted a small-scale, exploratory study. Our findings suggested that SGLT2-i therapy may improve oxidative stress and endothelial function, so we plan to investigate more patients and conduct sub-analysis of factors such as sex in the future. Some of the patients discontinued treatment during this study, which could potentially have influenced cardiovascular endpoints such as PWV, UCG, and RAAS. Therefore, this possibility should be assessed by a future study in a larger number of patients. Finally, all of our subjects were Japanese and it is unknown whether there are ethnic differences in the efficacy of SGLT2-is, suggesting the need to investigate other ethnic groups in the future.

## Conclusion

Treatment with canagliflozin demonstrated cardiac and renal protective effects, as well as improving oxidative stress, diastolic dysfunction, and endothelial function. We will continue to follow-up patients for a longer period. This drug was effective in patients with HFpEF and could potentially become first-line therapy for HFpEF patients with diabetes.

## Data Availability

Data sharing not applicable to this article as no datasets were generated or analyzed during the current study.

## Abbreviations

SGLT2-i: sodium–glucose cotransporter-2 inhibitor
HbA1c: hemoglobin A1c
eGFR: estimated glomerular filtration rate
CT: computed tomography
BW: body weight
BMI: body mass index
CPR: C-peptide immunoreactivity
BUN: blood urea nitrogen
sCr: serum creatinine
U-alb: urinary albumin excretion
T-cho: total cholesterol
LDL: low-density lipoprotein cholesterol
HDL: high-density lipoprotein cholesterol
TG: triglyceride
RLP-cho: remnant-like particles cholesterol
EPA/AA ratio: eicosapentaenoic acid/arachidonic acid ratio
Hb: hemoglobin
Ht: hematocrit
ANP: atrial natriuretic peptide
BNP: brain natriuretic peptide
Ox-LDL: oxidized low density lipoprotein
hs-CRP: high-sensitivity C-reactive protein
renin: plasma renin activity
PWV: pulse wave velocity
FMD: flow-mediated dilation
LVDd: left ventricular end-diastolic diameter
LVDs: left ventricular end-systolic diameter
EF: ejection fraction
%FS: percent fractional shortening (%FS)
LVMI: left ventricular mass index
HFrEF: heart failure with reduced ejection fraction
HFpEF: heart failure with preserved ejection fraction
RAAS: renin–angiotensin–aldosterone system
ACE-i: angiotensin-converting enzyme inhibitor
ARB: angiotensin II receptor blocker
